# ADDRESSING THE CHALLENGES OF MIPS WITHIN THE GWEP PROGRAM: A CALL TO CO-CREATE MEANINGFUL WAYS TO SHOW IMPACT

**DOI:** 10.1093/geroni/igad104.1703

**Published:** 2023-12-21

**Authors:** Anna Faul

**Affiliations:** University of Louisville, Louisville, Kentucky, United States

## Abstract

To address the challenges that unique challenges that the GWEP programs had in collecting and using MIPS data to show meaningful and quality work with older adult patients, we created a collaboration between NAGEC and the GWEP MIPS workgroup. Our primary goal was to develop a collective understanding of how GWEPs perceived MIPS as main outcomes. To better understand the collective challenges, a survey was created to examine the diversity in the sites across all GWEPs where MIPS data was collected and to quantify the types of challenges that GWEPs faced in collecting this type of patient data. From this survey we found the following issues were most prominent: 1.) Most GWEPs had primary care sites that had a difficult time pulling date from their electronic health records; 2.) Providers at participating primary care sites struggled to enter MIPS datapoints into the EHRs; 3.) Some of the participating primary sites did not collect MIPS data; 4.) Some of the participating primary care sites do not have the time to provide us with MIPS data in a timely fashion; and 5.) Some of our participating primary care sites are different than the traditional primary care sites (eg. skilled nursing facility, hospice, emergency departments), making MIPS measures irrelevant. From this survey, we were able to develop a solution focused statement to present to HRSA and to start the conversation about the difficulties and challenges in collecting MIPS data.

